# Supplementary material for: Comparative multiomics analysis of cell physiological state after culture in a basket bioreactor
Source: Sci Rep. 2022 Nov 23;12:20161. doi: 10.1038/s41598-022-24687-4 (PMC9686226; doi:10.1038/s41598-022-24687-4)
Supplement: Supplementary file 1 — Supplementary Information 1. [file 41598_2022_24687_MOESM1_ESM.zip › raw data/Transcriptomic raw data/6.Enrichment/2.KEGG/TE1vsTC1/ALL/src/csab04024.html]

csab04024


- csab:103224900

- Down regulated genes

103224900(-1.42998371610984)

- csab:103238835

- Up regulated genes

103238835(1.31802566292189)

- csab:103223064

- Down regulated genes

103223064(-1.75020224611862)

- csab:103222156

- Up regulated genes

103222156(1.83501644039466)

Close
